# Methanol extract of *Inonotus obliquus* improves type 2 diabetes mellitus through modifying intestinal flora

**DOI:** 10.3389/fendo.2022.1103972

**Published:** 2023-01-06

**Authors:** Xuewei Ye, Kefei Wu, Langyu Xu, Yingxin Cen, Jiahui Ni, Junyao Chen, Wenxin Zheng, Wei Liu

**Affiliations:** ^1^ Key Laboratory of Pollution Exposure and Health Intervention of Zhejiang Province, Department of Basic Medical Sciences, Shulan International Medical College, Zhejiang Shuren University, Hangzhou, China; ^2^ Institute of Plant Protection and Microbiology, Zhejiang Academy of Agricultural Sciences, Hangzhou, China

**Keywords:** *Inonotus obliquus*, T2DM, db/db mice, intestinal flora, SCFAs

## Abstract

Type 2 diabetes mellitus (T2DM) poses a significant risk to human health. Previous research demonstrated that *Inonotus obliquus* possesses good hypolipidemic, anti-inflammatory, and anti-tumor properties. In this research, we aim to investigate the potential treatment outcomes of *Inonotus obliquus* for T2DM and discuss its favourable influences on the intestinal flora. The chemical composition of *Inonotus obliquus* methanol extracts (IO) was analyzed by ultra-high-performance liquid chromatography-Q extractive-mass spectrometry. IO significantly improved the blood glucose level, blood lipid level, and inflammatory factor level in T2DM mice, and effectively alleviated the morphological changes of colon, liver and renal. Acetic acid, propionic acid, and butyric acid levels in the feces of the IO group were restored. 16S rRNA gene sequencing revealed that the intestinal flora composition of mice in the IO group was significantly modulated. *Inonotus obliquus* showed significant hypoglycemic and hypolipidemic effects with evident anti-inflammatory activity and improved the morphological structure of various organs and cells. *Inonotus obliquus* increased the levels of short-chain fatty acids in the environment by increasing the population of certain bacteria that produce acid, such as *Alistipes* and *Akkermansia*, which are beneficial to improve intestinal flora disorders and maintain intestinal flora homeostasis. Meanwhile, *Inonotus obliquus* further alleviated T2DM symptoms in db/db mice by down-regulating the high number of microorganisms that are dangerous, such as *Proteobacteria* and *Rikenellaceae_RC9_gut_group* and up-regulating the abundance of beneficial bacteria such as *Odoribacter* and *Rikenella*. Therefore, this study provides a new perspective for the treatment of T2DM by demonstrating that drug and food homologous active substances could relieve inflammation *via* regulating intestinal flora.

## 1 Introduction

Type 2 diabetes mellitus (T2DM), a chronic metabolic condition, is characterized by hyperglycemia, dyslipidemia, and impaired glucose tolerance, which can lead to a variety of chronic complications including diabetic nephropathy, cardiovascular disease, retinopathy, neuropathy, significantly lower patients’ quality of life and so on (1–3). With unhealthy lifestyles and dietary habits, the prevalence of diabetes has increased remarkably and become a public health problem that seriously threatens human health (4). A strong correlation between intestinal flora and diabetes began to reveal. Recent studies have shown that the intestinal flora plays an important role in maintaining gut homeostasis, especially in the regulation of inflammation (5). Through the interaction with dietary components, it affects intestinal permeability, glucose homeostasis, insulin sensitivity, glucose lipid metabolism and so on (6–8). Therefore, it is of great clinical significance and prospect to explore safer and more effective natural anti-diabetic drugs to regulate the intestinal flora of diabetic patients (9–11).

*Inonotus obliquus*, an edible fungus belongs to the genus *Fusarium* of the family Polyporaceae, is a valuable medicinal fungus with anti-aging, hypolipidemic, antitumor, anti-inflammatory, and anti-bacterial activities (12). *Inonotus obliquus* polysaccharide enhanced glucose tolerance and insulin sensitivity in T2DM mice, and had a significant hypoglycemic effect on T2DM mice. In addition, *Inonotus obliquus* shows a protective effect on the kidney of mice (13, 14). Previously, *Inonotus obliquus* has been shown to enhance glucose tolerance and insulin sensitivity in T2DM mice and improve hyperglycemic symptoms in type 2 diabetes patients (15). In addition, it can alleviate and protect kidney damage in patients with diabetic end-stage renal disease (16, 17). A recent study showed that a polysaccharide from *Inonotus obliquus* improved intestinal barrier dysfunction in mice with type 2 diabetes by upregating firmicutes abundance, inhibiting bacteroides levels and production of pro-inflammatory factors (18). However, the mechanism and efficacy of *Inonotus obliquus* to improve other symptoms of type 2 diabetes mice by altering intestinal microbiome remain unclear. Thus, this study investigated the beneficial therapeutic effects of *Inonotus obliquus* on intestinal flora dysbiosis in db/db mice. We found that *Inonotus obliquus* displayed consistent hypoglycemic effects, reduced body weight, improved lipid metabolism disorders, alleviated inflammation and the degree of lesions in different organs, and regulated the makeup and metabolism of the intestinal flora in db/db mice.

## 2 Materials and methods

### 2.1 Extraction and principal component analysis of *Inonotus obliquus*

*Inonotus obliquus* was provided by Ji ‘an Tianhe Ginseng Antler Products Co., LTD (222894S-2019).

The air-dried *Inonotus obliquus* were pulverized for extraction. The ground powder of *Inonotus obliquus* (1 *kg*) was extracted with 1500 mL of methanol by sonication for 90 min, repeating 3 times. After filtration, all extracts were combined and distilled under reduced pressure. The *Inonotus obliquus* methanol extracts (IO) was concentrated to dryness *in vacuo* to provide samples for biological testing (19).

ACQUITY UPLC^®^ HSS T3 column (2.1 × 150 mm, 1.8 µm) was used for ultra-high-performance liquid chromatography-Q extractive-mass spectrometry (UHPLC-QE-MS) analysis. Positive ions (0.1% formic acid water (C) - 0.1% formic acid acetonitrile (D)); negative ions (5 mM ammonium formate water (A) - acetonitrile (B)) were used as mobile phases. The gradient elution program was set as follows: 0~1 min, 2% B/D; 1~9 min, 2~50% B/D; 9~12 min, 50~98% B/D; 12~13.5 min, 98% B/D; 13.5~14 min, 98%~2% B/D; 14~20 min, 2% D - positive mode (14~17 min, 2% B - negative mode). The Electrospray Ionization Mass Spectrometry (ESI-MS) source conditions were set as sheath gas 30 arb, auxiliary gas 10 arb, capillary temperature 325°C, resolution 60,000 positive and negative ionization mode, spray voltage 3.50 kV (positive), spray voltage 2.50 kV (negative).

### 2.2 Animal experiments

A total of 24 C57BKS-db mice (db/db, male, eight-week-old, 45 ± 5 g) and eight C57/BKS mice (wild type, male, eight-week-old, 25 ± 5 g) were used. Animals (SPF grade) were provided by the Jiangsu GemPharmatech Co., LTD and were kept on adaptive feeding for a week. The protocol was approved by the Committee on the Ethics of Animal Experiments of the Zhejiang Academy of Agricultural Sciences. All mice were kept in an SPF-class animal house of the Zhejiang Academy of Agricultural Sciences, fed with conventional feed ad libitum, and a 16-hour artificial LED light cycle is maintained indoors at a constant temperature of 20 °C and relative humidity of 55%. Mice with random Blood sugar level more than 11.1 mmol/L on various days were chosen at random for the experimental group after blood was drawn from the tail vein over the course of three days. According to blood glucose and body weight, the DB group was created by randomly selecting db/db mice (model, saline), the DM group (metformin hydrochloride 200 mg/kg, positive control), and the IO group (IO intervention, 600 mg/kg) (12, 20, 21). The solvent used for intragastric administration was 0.9% normal saline.There were eight mice in each group. Eight C57/BKS (Wild Type) mice were set as the K group (blank control, saline). The administration was fixed at the same time, once every day, for eight weeks of continuous intervention. Fasting blood glucose, OGTT test, and mice feces were collected weekly. After blood sampling, animals were sacrificed by cervical dislocation at the end of the fourth and eighth weeks. The tissues from the kidney, pancreas, and liver were promptly separated, removed, wiped on filter paper, and weighed on an electronic balance.The organ weight coefficients were calculated according to the formula: Organ weight coefficient = organ weight (g)/body weight (g) (22). The colon, liver, and kidney tissues were taken and fixed in 4% paraformaldehyde for histopathological examination. The feces, blood samples, and remaining tissues were frozen at -80 °C.

### 2.3 Biochemical testing

#### 2.3.1 Analysis of fasting blood glucose and oral glucose tolerance test

The OGTT test was performed after weekly administration. All mice fasted without water the night before the experiment. After 12 h, FBG was determined as 0 min blood glucose. After weighing them, mice were given a glucose solution at a rate of 2 mg/kg *via* gavage. Following glucose loading, blood glucose was taken at 30, 60, 90, and 120 min. The area under the curve (AUC) was then computed (23).

#### 2.3.2 The detection of inflammatory factors and blood lipid levels

Blood was extracted from the ocular vein of mice and rested for 2 h. After that, it spent 20 min in a water bath at 37°C. After centrifuging the blood at a temperature of 4 degrees Celsius for 10 min at a rate of 3000 revolutions per min, the serum was collected (24).

ELISA kit was used to test inflammatory factors purchased from Boster Biological Technology Co., LTD. Serum tumor necrosis factor-α (TNF-α, EK0527), interleukin-1β (IL-1β, PROTP10749), interleukin-6 (IL-6, EK0411), and interleukin-10 (IL-10, EK0417) assays were performed according to Boster manufacturer’s instructions. Add 100 μL of samples and standards to each well, and react at 37°C for 90 min. Add 100 μL of biotin-labeled antibody to each well, react at 37°C for 60 min, and wash with 0.01M TBS three times after the reaction. Add 100 μL ABC to each well, react at 37°C for 30 min, and wash 5 times with 0.01M TBS again. TMB was reacted at 37°C in the dark for 30 min, and finally TMB stop solution was added for reading.

The blood lipid levels were detected according to the kits purchased from Purebio Biotechnology Co., LTD. Serum Total Cholesterol (TC, CH01, 20210622), Triacylglycerol (TG,TG01, 20210618), High-density lipoprotein cholesterol (HDL-C, HL01, 20210522)and Low-Density Lipoprotein Cholesterol(LDL-C, LDL01, 20210428) concentrations were measured according to the manufacturer’s instructions of GPO-PAP method, CHOD-PAP method, direct method-selective inhibition method, direct method-surfactant scavenging method were used for detection.

### 2.4 Histopathological analysis and pathological histology score

After embedding, sectioning, and H&E staining, the histomorphology of the liver, colon, and kidney tissues was observed under the microscope. The pathological tissue score for each organ was based on the relevant literature (6, 25–27) and the scoring criteria are showed in **Tables S1-S4**. The kidney pathological score was calculated as the sum of the glomerular and tubular scores.

### 2.5 Analysis of intestinal flora and metabolism

#### 2.5.1 Short-chain fatty acids (SCFAs) analysis

We determined four major SCFAs: acetic acid, propionic acid, butyric acid, and isobutyric acid. Feces were placed in a centrifuge tube with PBS buffer (pH 7.4) at a ratio of 1:10 to dissolve the samples, and the mixture was vortexed and mixed thoroughly. Filter the supernatant (4°C, 10 000 r/min, 5 min) through 0.45 μm filter paper into a clean centrifuge tube. The filtrate was acidified with crotonic acid at 5:1 for 24 h, and then the SCFAs were determined by gas chromatography (GC) (28). GC (Shimadzu, Japan) with DB-FFAP column (0.32 mm × 30 m × 0.5 μm, Agilent Technologies, USA) was used to quantify the SCFAs. The operation condition was as follows: the flow rate of nitrogen carrier gas: 19.0 mL/min; split ratio: 1:10, the temperature of both detector and injection port: 250 °C. Crotonic acid was used as internal standard.

#### 2.5.2 DNA extraction and 16S rRNA gene sequencing

Genomic DNA was extracted from fecal samples with TruSeq Nano DNA LT Sample Preparation Kit (Illumina, USA) following the kit protocol. The bacterial 16S rRNA gene was amplified using a 343F/798R primer set (343 F: TACGGRAGGCAGCAG; 798 R: AGGGTATCTAATCCT) targeting the V3-V4 region. PCR products are detected using electrophoresis and purified using magnetic beads. The DNA concentration was detected using agarose gel electrophoresis and NanoDrop2000, and the 16S rRNA gene targeting the V3-V4 region was amplified by PCR with Tks Gflex DNA polymerase (Tks Gflex DNA Polymerase, Takara, China). Then the PCR product was detected by electrophoresis, purified by magnetic beads and then used as a template for the second round of PCR to perform PCR amplification, electrophoresis detection and magnetic bead purification again. The PCR product was tested for Qubit concentration immediately after purification. According to the concentration of the same amount of polyculture, it was sequenced on the machine. The arduous sequencing and data analysis of 16S rRNA was carried out in Shanghai Ouyi Biological Co., Ltd. Using the Vsearch (29) software, and according to the sequence similarity, the sequences were classified into multiple OUTs, and the sequence similarity of the OUTs of a unit was greater than or equal to 97%. Quantitative Insights Into Microbial Ecology (QIIME) software was used to select sample sequences from generated and deduplicated sequences for each Optical Transformation Unit (OTU) and annotated against the Silva (version 138) database with confidence intervals greater than 0.7 Annotations result. Alpha diversity analysis (Chao1, Shannon, Simpson and Good’s Coverage) and Beta diversity analysis (PCoA, NMDS) were analyzed using QIIME software. In addition, correlation analysis, LDA Effect Size (LEfSe) analysis, and linear discriminant analysis (LDA) were used to calculate and determine difference groups using correlation analysis tools and LEfSe tools, respectively. The flora prediction was analyzed using STAMP software.

### 2.6 Statistical analysis

The mean SD is used to express all experimental data. For normally distributed data, paired t test was performed between two groups, and one-wayANOVA followed by LSD test was performed between multiple groups. *P* < 0.05 was regarded as statistically significant. All data were statistically analyzed using SPSS 22.0 software. 16S rRNA analysis uses Silva (version138) database alignment, whereas species alignment annotation uses an RDP classifier (30) (confidence threshold was 70%).

## 3 Results

### 3.1 Identification of the chemical composition of *Inonotus obliquus*

The components of *Inonotus obliquus* were analyzed and identified using UHPLC-QE-MS. The Traditional Chinese Medicine Systems Pharmacology (TCMSP) and Analysis Platform databases were accessed to screen the bioactive compounds based on oral bioavailability % (OB%) and drug-likeness (DL) content (OB% > 30 and DL > 0.18). Fourteen active compounds were identified after the screening, including six flavonoids, one coumarin, one organic acid, and six other compounds (Anthracenes, beta-Carotene, Endogenous Metabolites, Steroids and steroid derivatives, Fatty Acyls, Carboxylic acids and derivatives). **Table 1** shows the mass-to-charge ratios, types and identified compounds of the active compounds.

**Table 1:** Characterization of chemical constituents of *Inonotus obliquus*.

No.	RT (min)	m/z	Type	Formula	OB (%)	DL	Identification	Class
1	5.56	303.104	[M+H]^+^	C_16_H_14_O**_6_**	70.31	0.27	Hesperetin	Flavonoids
2	5.94	301.001	[M-H]^-^	C_14_H_6_O_8_	43.06	0.43	Ellagic acid	Tannins
3	6.71	291.084	[M+H]^+^	C_15_H_14_O_6_	48.96	0.24	Epicatechin	Flavonoids
4	7.87	269.046	[M-H]^-^	C_15_H_10_O_5_	33.52	0.21	Baicalein	Flavonoids
5	8.29	301.035	[M-H]^-^	C_15_H10O_7_	46.23	0.27	Morin	Flavonoids
6	9.68	447.089	[M+H]^+^	C_21_H_18_O_11_	40.12	0.75	Baicalin	Flavonoids
7	9.95	283.022	[M-H]^-^	C_15_H_8_O_6_	47.07	0.28	Rhein	Anthracenes
8	10.64	301.037	[M-H]^-^	C_15_H_10_O_7_	46.43	0.28	Quercetin	Flavonoids
9	11.18	536.183	[M]^+^	C_40_H5_6_	37.18	0.58	beta-Carotene	Terpenoids and Glycosides
10	11.27	271.094	[M+H]^+^	C_16_H_14_O_4_	34.55	0.22	Imperatorin	Coumarins and derivatives
11	11.38	272.062	[M+H]^+^	C_15_H_11_O_5_	37.99	0.21	Pelargonidin	Endogenous Metabolites
12	13.28	393.301	[M+H]^+^	C_24_H_40_O_4_	40.72	0.68	Deoxycholic acid	Steroids and steroid derivatives
13	13.7	305.249	[M+H]^+^	C_20_H_32_O_2_	45.57	0.2	Arachidonic acid	Fatty Acyls
14	13.71	439.361	[M+H-H_2_O]^+^	C_30_H_48_O_3_	55.38	0.78	Betulinic acid	Carboxylic acids and derivatives

### 3.2 Effects of *Inonotus obliquus* on FBG and OGTT in db/db mice

**Figure 1:**
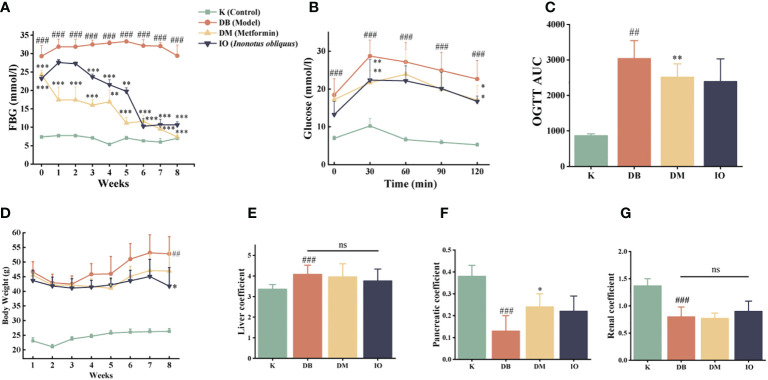
Influence of blood glucose utilization and organ coefficient *Inonotus obliquus* and metformin reduced blood glucose in db/db mice during the intervention period. FBG **(A)** and OGTT **(B, C)**. *Inonotus obliquus* and metformin exerted a weight loss effect **(D)**. *Inonotus obliquus* and metformin on liver coefficient **(E)**, pancreatic coefficient **(F)** and renal coefficient **(G)** in db/db mice. Data were expressed as the mean ± SD. ^##^ *p* < 0.01 vs. K, ^###^ *p* < 0.001 vs. K, *p < 0.05 vs.DB, **p < 0.01vs. DB, ***p < 0.001vs. DB.

Prior to pharmacological intervention, the mice in the DB, DM, and IO groups had substantially lower fasting blood glucose levels than the mice in the K group (*p* < 0.001) (**Figure 1A**). After three-week drug interventions, the DM and IO groups had much less fasting blood glucose than DB groups. (*p* < 0.001). Blood glucose levels in the IO group leveled off after six weeks and were substantially different from those in the DB group (*p* < 0.001). The mice in each group had elevated glucose levels following oral glucose injection, which persisted for 0 to 30 min. After 30–60 min, the glucose level of mice in each group gradually decreased. In contrast to the K group, the blood glucose and AUC of the DB group at each time point from 0–120 min were notably higher than those in the K group (*p* < 0.01). Compared with the DB group, the mice in the IO group showed a decreasing trend in blood glucose and AUC at each time point, including an evident decrease in blood glucose level at 30 min (*p* < 0.01), a remarkable decrease at 120 min (*p* < 0.05) and restored basal level (**Figures 1B, C**).

### 3.3 Effects of *Inonotus obliquus* on the changes of body weight and organ coefficients in db/db mice

The body weights of the DB, DM, and IO groups were comparable at the start of the trial and were noticeably greater than those of the K group (*p <*0.01). By the conclusion of the eighth week, the IO group of mice were much lighter than the DB group of mice in terms of body weight (*p* < 0.05) (**Figure 1D**). The liver organ coefficients of mice in the DB group rose considerably (*p* < 0.01) as compared to the K group (*p* < 0.01). However, the renal and pancreatic organ coefficients, substantially decreased (*p* < 0.01). Mice in the DM group had higher pancreatic organ coefficients than mice in the DB group (*p* < 0.05), and the ratio of liver weight to total weight reduced in mice in the IO group, while the renal and pancreatic weights tended to increase, all of which were closer to those in the K group (**Figures 1E–G**).

### 3.4 Effects of *Inonotus obliquus* on blood lipid levels

**Figure 2:**
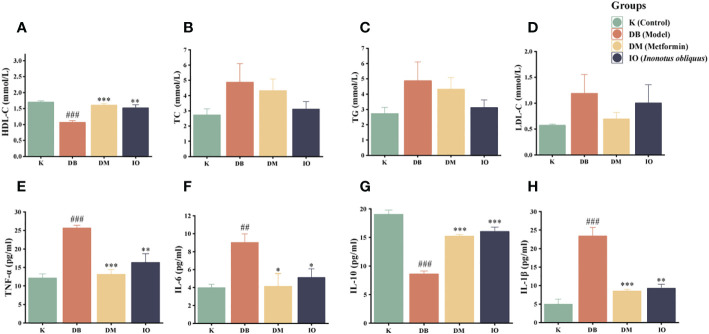
Effects of lipid metabolism and inflammatory cytokines. HDL-C **(A)**, TC **(B)**, TG **(C)** and LDL-C **(D)**. TNF-α **(E)**, IL-6 **(F)**, IL-10 **(G)** and IL-1β **(H)** inflammatory cytokines in the plasma of db/db mice. Data were expressed as the mean ± SD. ^###^ *p* < 0.001 vs. K, *p < 0.05 vs. DB, **p < 0.01vs. DB, ***p < 0.001 vs. DB.

As shown in **Figures 2A–D**, the mice in the DB group exhibited hyperlipidemia with decreased HDL-C levels (*p* < 0.001) and increased LDL-C, TC, and TG levels. Compared with the mice in the DB group, the mice in the IO group showed a higher HDL-C (*p* < 0.01) and lower LDL-C, TC, and TG levels.

### 3.5 Effects of *Inonotus obliquus* on inflammatory factor

The inflammatory factors TNF-α (*p* < 0.001), IL-1β (*p* < 0.001), and IL-6 (*p* < 0.01) were increased in the DB group mice compared with the K group, although IL-10 was severely diminished (*p* < 0.001). Compared with the DB group, the expression levels of TNF-α and IL-1β were considerably decreased in the IO group (*p* < 0.01) and IL-6 was considerably decreased (*p* < 0.05) which compare to DB group. IL-10 expressed at a considerably greater level (*p* < 0.001). These data are illustrated in **Figures 2E–H**.

### 3.6 Effects of *Inonotus obliquus* on histomorphological changes in organs

**Figure 3:**
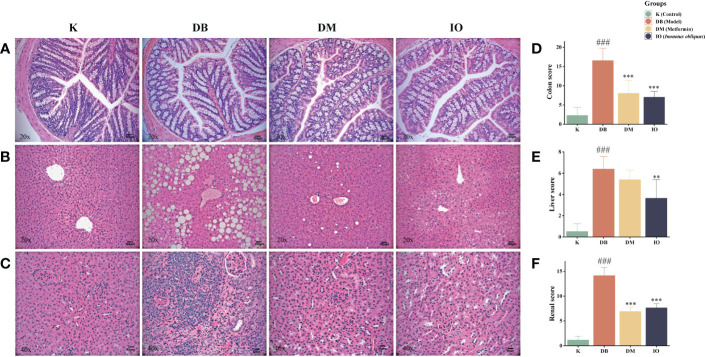
Histopathologic changes after 8 weeks of drug intervention. **(A)** Representative images of morphological changes in colon tissue. **(B)** Representative images of morphological changes in liver tissue. **(C)** Representative images of morphological changes in kidney tissue. **(D)** Histogram of colon tissue score. **(E)** Histogram of liver tissue score. **(F)** Histogram of renal tissue score. Data were expressed as the mean ± SD. ###p < 0.001 vs. K, **p < 0.01 vs. DB, ***p < 0.001 vs. DB.

Histopathological damage of the colon, liver, and renal was assessed by HE staining (**Figures 3A, D**). In K group, the mucosal epithelium of colonic tissues was intact, liver tissues were free of fibrosis, glomeruli were free of hypertrophy, and all tissues were free of inflammatory cell infiltrations. In the DB group of mice, colonic submucosa edema was seen, with a large number of cup cells disappearing, liver tissue steatosis, edema and fibrosis were severe, glomerular hypertrophy and capillary basement membrane thickening were seen, and all tissues were infiltrated with a large number of inflammatory cells. The organ morphology of the T2DM mice and the healthy normal mice were very dissimilar. All histopathological scores were greatly increased in the T2DM mice compared with the healthy normal mice (*p* < 0.01). Compared with the T2DM mice, no loss of cup cells was seen in the colonic tissues of mice in the *Inonotus obliquus* and metformin-gavaged mice, the degree of crypt damage was reduced, and the colonic histopathological score was extremely significantly lower (*p* < 0.001) (**Figures 3A-D**). The liver fat degeneration and fibrosis were reduced. In the IO group, the liver tissue score was considerably lower (*p* < 0.01) (**Figures 3B, E**); There was a more typical glomerular shape, no cystic stenosis, and little inflammatory cell infiltration across all tissues; substantially less renal tissue was scored in the IO group (*p* < 0.05) (**Figures 3C, F**).

### 3.7 Effects of *Inonotus obliquus* on intestinal flora

#### 3.7.1 Alpha and beta diversity analysis

**Figure 4:**
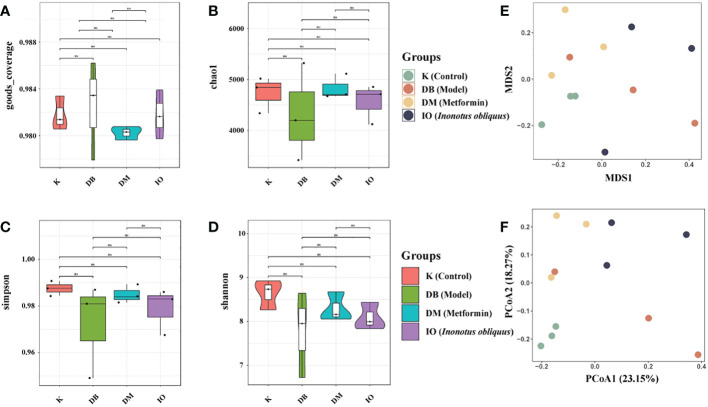
Alpha and Beta diversity analysis. Alpha diversity analysis indicates the degree of species diversity within a single biological environment. Beta diversity analysis clearly shows a clear separation between DB groups and IO groups. **(A)** Goods coverage; **(B)** Chao1; **(C)** Simpson; **(D)** Shannon. **(E)** NMDS1 and NMDS2 are two sorting axes, each point in the figure represents a sample, the same color is the same group, the IO group is separated from the module DB group; **(F)** Weighted UniFrac PCoA map based on OTU abundance. NS, non-significant, p>0.05. The first two principal coordinates (PC1 and PC2) of PCoA from weighted UniFrac were plotted for each sample to assess similarity between samples and groups.

Alpha diversity analysis indicates the degree of species diversity within a single biological environment, which is usually measured using two indices: species richness and species evenness. Species richness is more responsive to the Shannon index, but species evenness is more sensitive to the Simpson index.The chao1 index reflects the community species richness; the higher the value, the higher the species richness. Good’s Coverage reflects the OTU coverage of the sample (31). As shown in **Figures 4A–D**, the Good’s Coverage values of the samples were above 97%, indicating that the sequences present in the samples were detected. In this study, we found that the intestinal flora richness and homogeneity of mice in the DB, DM and IO groups were reduced compared to the K group. In contrast to the DB group, the DM and IO groups’ intestinal flora was more diverse and homogeneous. The differences in non-metric multi-dimensional scaling (NMDS) and principal co-ordinates analysis (PCoA) matrix scores (**Figures 4E-F**) indicated that all four groups of mice had different intestinal microbiota. The intergroup distance between the mice in groups K and DB was farther, showing that the mice in group DB significantly changed intestinal microbiota composition. The mice in groups IO and DM showed closer intergroup distance and were separated from those in group DB.

#### 3.7.2 Changes in flora composition at the phylum and genus levels

**Figure 5:**
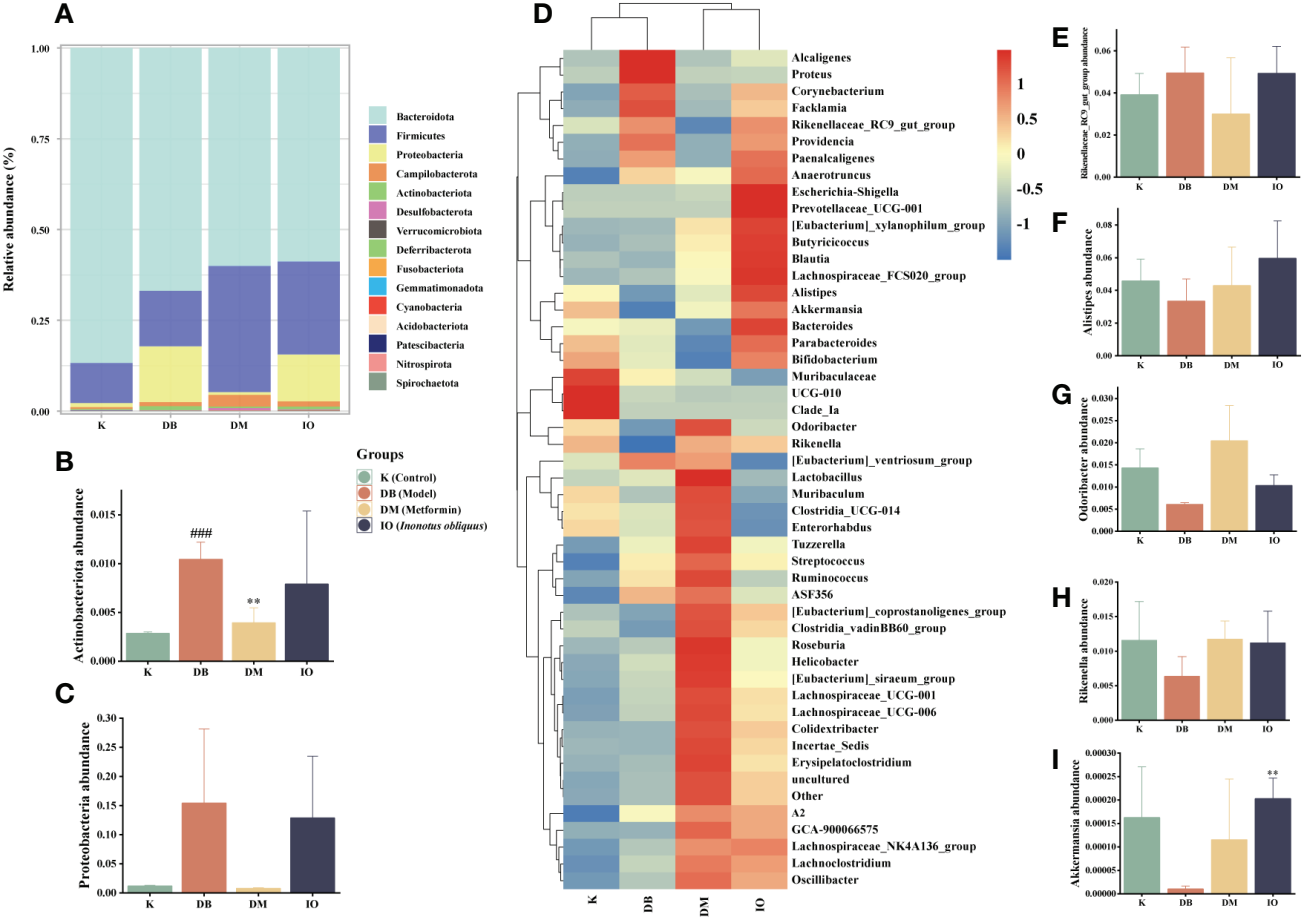
Structural changes of intestinal flora. Intestinal flora species abundance at the phylum and genus levels. **(A)** The relative abundance of microbial species at the phylum level in the feces of mice, **(B)** *Actinobacteriota*, **(C)** *Proteobacteria*. **(D)** The relative abundance of microbial species at the genus level in the feces of mice. **(E)** *Rikenellaceae_RC9_gut_group*, **(F)** *Alistipes*, **(G)** *Odoribacter*, **(H)** *Rikenella*, **(I)** *Akkermansia*. Data were expressed as the mean ± SD. ^###^ *p* < 0.001 vs. K, **p < 0.01 vs. DB.

The relative abundance values of microorganisms in each group of mice fecal samples were calculated by analyzing the structural distribution of microbial colonies in the samples based on the biotaxonomic level. At the level of the phylum (**Figure 5A**), the abundance of the same species in different groups was indicated, visually reflecting the variation in colony abundance. The groups were mainly composed of *Bacteroidetes*, *Firmicutes*, *Campilobacterota*, *Actinobacteriota*, and *Desulfobacterota*. Among them, *Firmicutes* and *Bacteroidetes* comprised a more significant proportion. The abundance of *Proteobacteria* (**Figure 5B**) and *Actinobacteriota* (**Figure 5C**) was significantly higher in the DB group than in the K group. The abundance of *Proteobacteria* and *Actinobacteria* was decreased in the DM and IO groups compared to the DB group. Compared with the K group at the genus level (**Figure 5D**), the DB group had a higher relative abundance of the *Rikenellaceae_RC9_gut_group* (**Figure 5E**) than the K group. *Alistipes* (**Figure 5F**), *Odoribacter* (**Figure 5G**), *Rikenella* (**Figure 5H**), and *Akkermansia* (**Figure 5I**) decreased in relative abundance.

#### 3.7.3 LEfSe analysis

**Figure 6:**
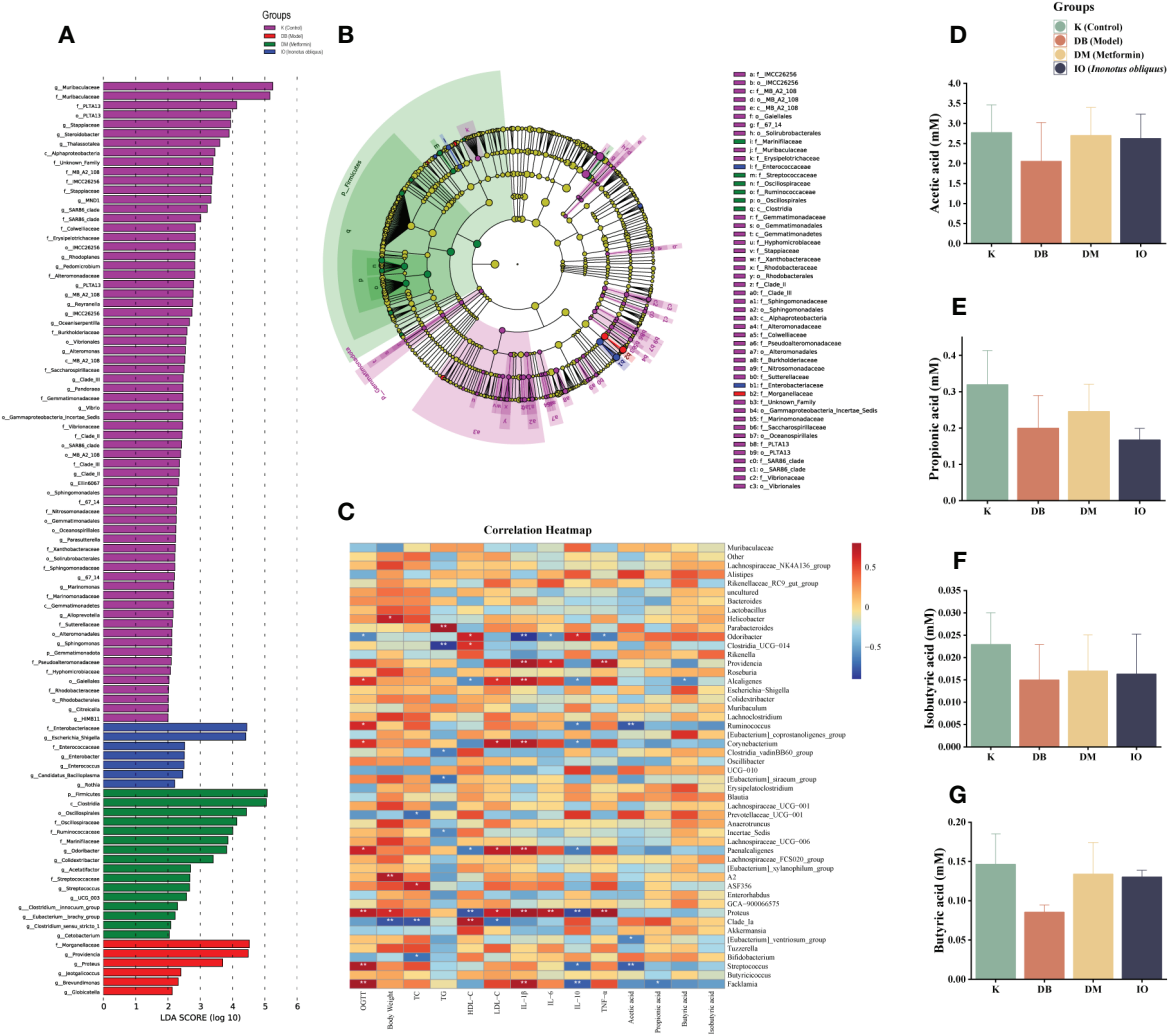
LEfSe, SCFAs leves, and correlation analysis. **(A)** Levels LEfSe analyzed gut microbiota that differed significantly among groups. Histogram of LDA scores of fecal 16S rRNA sequences from phylum level to genus level with LDA score >3; **(B)** The inner-to-outer radiating circle represents the evolutionary cladogram from phylum to genus; **(C)** Heat map of the relationship between intestinal microbiota at species level and diabetes-related indexes (The blue and red represent negative and positive correlation, respectively. *p < 0.05; **p < 0.01); *Inonotus obliquus* and metformin can change the composition of short chain fatty acids (SCFAs) in the stool of mice. *Inonotus obliquus* and metformin could restore fecal short-chain fatty acid content in db/db mice. *Inonotus obliquus* alters fecal short-chain fatty acids (SCFAs) levels of **(D)** acetate acid, **(E)** propionate acid, **(F)** butyrate acid and **(G)** isobutyrate acid.

LDA discriminant bar graphs (LDA score > 3) and evolutionary branching graphs (**Figures 6A, B**) were done at the genus level. According to the findings, in the DB group, Providencia and Proteus were the dominant flora; in the DM group, *Firmicutes*, *Oscillospiraceae*, *Ruminococcaceae*, and *Odoribacter* were the dominant, and *Enterobacteriaceae*, *Escherichia*, and *Shigella* were dominant in IO group.

#### 3.7.4 Effect of *Inonotus obliquus* on SCFAs

Butyric acid, acetic acid, and propionic acid are the three most common SCFAs, and they are the major end products of dietary fiber fermentation by gut bacteria. **Figures 6C–F** showed that the acetic acid, propionic acid, butyric acid, and isobutyric acid concentrations in the DB group were lower than those in the K group. However, the concentration of acetic acid, propionic acid, butyric acid, and isobutyric acid was increased in the DM and IO groups compared with that of the DB group.

#### 3.7.5 Correlation analysis with environmental factors

**Figure 6G** of the correlation study revealed that *Odoribacter* was inversely connected with OGTT, TNF-α, IL-6, IL-1β, and other physiological and biochemical markers of T2DM (*p* < 0.05), while *Proteus* was favorably correlated with IL-1β, IL-6, and TNF-α (*p* < 0.01) and strongly correlated with TNF (*p* < 0.001).

#### 3.7.6 Colony function prediction

**Figure 7:**
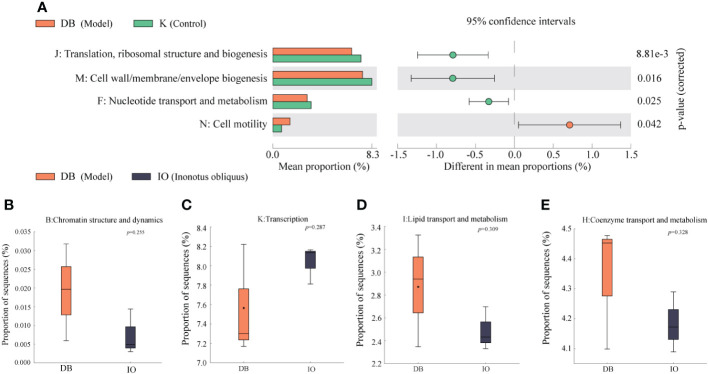
Function prediction. **(A)** Analysis of the differences in the prediction of intestinal flora function based on COG pathway between DB group and K group. Analysis of the differences between the DB group and the IO group in the functions of **(B)** Chromatin structure and dynamics, **(C)** Transcription, **(D)** Lipid transport and metabolism, and **(E)** Coenzyme transport and metabolism.

As shown in **Figure 7**, the protein function clusters predicted in this experiment were divided into 23 major groups according to the metabolic functions of the proteins. The annotation information of each functional level of mouse intestinal flora cluster of orthologous group (COG) was the same for each group, while the abundance information of each function differed between groups. In contrast to the mice in the K group, mice in the DB group had highly significant differences in COG function in J: Translation, ribosomal structure and biogenesis, M: Cell wall/membrane/envelope biogenesis, F: Nucleotide transport and metabolism and N: Cell motility (*p* < 0.01). Significant discrepancies were found between the IO and DB groups (*p* < 0.05): B: Chromatin structure and dynamics, K: Transcription, I: Lipid transport and metabolism and H: Coenzyme transport and metabolism.

## 4 Discussion

In this study, we found that *Inonotus obliquus* effectively reduced blood glucose, body weight, and lipids in db/db diabetic mice. It also had significant anti-inflammatory effects and inhibited the progression of lesions in various organs, indicating that the diabetic symptoms in mice were effectively improved. In addition, 16S rRNA gene sequencing of intestinal flora showed that *Inonotus obliquus* treatment could result in alterations in the overall composition and metabolism of intestinal flora in mice, which in turn improve the symptoms of T2DM.

### 4.1 Active substances in IO and its effect on improving diabetes

Using the UHPLC-QE-MS method, we studied the chemical composition of *Inonotus obliquus* and elucidated the major compounds. We identified 14 compounds, including steroids, flavonoids, coumarins, and organic acids. Epicatechin added to the mice diet had higher sensitivity to insulin than controls. Also, epicatechin inhibited gluconeogenesis in the liver, enhanced glycolysis, and promoted muscle glucose uptake by activating 5’AMP-activated protein kinase (AMPK) (32). Thus, by reducing blood glucose, epicatechin may have antidiabetic benefits. An important key characteristics of diabetes is obesity, and it has been demonstrated that β-carotene plays a crucial role in adipogenesis, lipolysis, insulin resistance, and other factors associated with T2DM (33). Thus, beta-carotene plays a role in mature adiposity, helps control lipid metabolism, regulates oxidative stress, and alleviates inflammation. Arachidonic acid is a potent inducer of insulin secretion, while its metabolites contribute differently to insulin resistance and play a crucial part in the onset and progression of prevalent disorders including diabetes and obesity (34). The anti-inflammatory activities of baicalein, baicalin, ellagic acid, and pelargonidin have all been reported (35–38). Additionally, morin might have effects that were anti-inflammatory, antidiabetic, and anticancer by modifying the activity of various enzymes (39). Hesperetin can exert anti-inflammatory activity by reducing pro-inflammatory cytokines and has antioxidant, hypolipidemic, and insulin-sensitizing properties that are therapeutic for various diseases and disorders (40). Quercetin exhibits sufficient anti-inflammatory activity to alleviate T2DM-induced inflammation, and it has been reported that quercetin ameliorates inflammation by downregulating nitric oxide synthase (NOS) expression and reducing TNF-α and nitric oxide production in visceral adipose tissue of obese rats (41, 42).

Imperatorin exerts its anti-inflammatory effect in macrophages at varying doses by inhibiting catalase, superoxide dismutase, and TNF-α (43, 44). In addition, some antitumor drugs also have anti-inflammatory activity (45), which might allow imperatorin to achieve antitumor effects by inhibiting macrophage inflammation. Rhein lowers inflammation by inhibiting the production of adhesion molecules on endothelial cells (ECAM) (46, 47). Thus, it is suggested that rhein has anti-inflammatory activity and improves the symptoms of T2DM. intestinal flora can convert deoxycholic acid to ursodeoxycholic acid (UDCA). UDCA has been found to reduce FBG and insulin concentrations (48). Furthermore, it has antioxidant, anti-inflammatory and cytoprotective effects and plays a significant function in lipid metabolism and intestinal barrier integrity (49). This suggests that deoxycholic acid can be modified to UDCA through intestinal flora metabolism and is able to lower blood glucose and have a positive effect on glucose tolerance while acting in the intestine to alleviate inflammation, protect the intestinal barrier and maintain intestinal microecological balance. In addition, it also has some anticancer potential (50), and because of its large content in *Inonotus obliquus*, it may have antitumor potential that warrants further research in the future (51). The above studies showed that *Inonotus obliquus* contains compounds with good hypoglycemic, lipid metabolism regulation, obesity improvement, and anti-inflammatory activities and has great antitumor potential, which needs further study (52).

### 4.2 The relationship between the improvement of diabetes and intestinal flora

Based on the ameliorative effect on diabetes, we performed 16S rRNA gene sequencing on feces samples and found that the effect of *Inonotus obliquus* on intestinal microbiota was on both the flora composition and the metabolism. Analyses of alpha diversity revealed that after treatment with *Inonotus obliquus*, the diversity and richness of the intestinal flora of mice in the IO group increased relative to the K group, which was similar to the results observed in the DM group. This suggested that *Inonotus obliquus* and metformin administration can significantly affect intestinal microbiota’s abundance and improve intestinal flora’s dysbiosis in db/db mice. PCoA and NMDS data revealed that IO affected the makeup of the intestinal flora. Compared to the K group, DB group had more *Proteobacteria* at the phylum level than IO group. It has been shown that *Proteobacteria* and TC levels exhibited a substantial positive link, as demonstrated by the present study results, which revealed an increase in TC levels among mice in the DB group and a decrease among mice in the IO group. It also demonstrated that blood glucose is positively correlated with *Proteobacteria*. In the current research, the OGTT level in the DB group rose, whereas the TC level in the IO group declined. The decrease of *Proteobacteria* and *Actinobacteria* in the IO group mice suggested that IO had some improvement effect on mice intestinal flora. IO may regulate glycolipid metabolism and improve glycolipid levels in db/db mice by regulating the abundance of *Proteobacteria* and *Actinobacteriota* flora. When compared to the K group, the relative abundance of the *Rikenellaceae_RC9_gut_group* gene was more abundant in the DB group whereas it was less abundant in the IO group. Another study showed that HFD might enhance the *Rikenellaceae_RC9_gut_group* (53). This suggested that IO may regulate abnormal lipid metabolism by regulating intestinal flora disorders and reducing the abundance of *Rikenellaceae_RC9_gut_group*, thereby improving the hyperlipidemia level in db/db mice.

### 4.3 Interconnections between microbiota and metabolites

Mice in the DB group had lower levels of *Alistipes*, *Odoribacter*, and *Rikenella* than mice in the K group, whereas mice in the IO group exhibited levels that were greater than those in the DB group. *Alistipes* metabolism could produce some acetic acid and propionic acid. The SCFAs findings revealed that acetic and propionic acid concentrations were higher in the DM and IO groups than in the DB group. By preventing neutrophil and macrophage production of pro-inflammatory cytokines, acetic acid and propionic acid salts can successfully reduce inflammation. It is suggested that intestinal flora metabolize *Inonotus obliquus* to produce acetic acid and propionic acid, which can reduce inflammation in db/db mice.

In addition, it has been demonstrated that *Alistipes* might have a preventive impact against certain diseases, including colitis, liver fibrosis, cancer immunotherapy, and cardiovascular disease. A decrease in its abundance has been associated with an increased recurrence of hepatic encephalopathy, which is consecutively associated with the progression of cirrhosis to a decompensated state (54). Our study found that the liver HE sections showed reduced liver fibrosis in the IO group of mice. It is suggested that *Inonotus obliquus* might improve the severity of liver lesions in db/db mice by increasing the abundance of *Alistipes* in the intestine. The environmental factor correlation analysis results showed that *Odoribacter* and OGTT, TNF-α, IL-6, IL-1β, and other physiological and biochemical correlates of T2DM were negatively correlated (*p* < 0.05). The above-mentioned study showed that abundant *Rikenella* and *Odoribacter* could simultaneously up-regulate OGTT, TNF-α, IL-6, and IL-1β and alleviate the symptoms of T2DM. We found that the relative abundance of *Akkermansia* was reduced in DB mice and elevated in the DM and IO groups compared to the K group, which is consistent with the previous studies (55, 56).

*Akkermansia*, a butyric acid-producing bacterium, decreased in numbers leading to mild inflammation in the intestine of diabetic mice. SCFAs outcomes showned that the mice in the DB group had lower butyric acid levels than those in the K group, whereas the mice in the IO group had higher butyric acid levels than those in the DB group. Butyric acid is essential in maintaining colonic epithelial homeostasis, mainly as an anti-inflammatory agent. Additionally, research has shown that the oral glucose insulin sensitivity (OGIS) model’s assessment of postprandial insulin sensitivity was directly connected with butyrate levels (57, 58). This suggests that *Inonotus obliquus* can improve the content of butyrate in db/db mice by increasing the abundance of *Akkermansia*. In this way, it exerts its anti-inflammatory and insulin sensitivity-enhancing effects. The predicted results of flora function suggested that differential flora may improve glucose homeostasis and insulin sensitivity in db/db mice by affecting functions such as E (amino acid transport and metabolism) and G (carbohydrate transport and metabolism). Furthermore, *Akkermansia* is essential for preserving the integrity of the mucin layer (59). The above findings suggested that IO can maintain the integrity of the mucin layer and reduce inflammation by regulating the butyrate content in the SCFAs and the abundance of *Akkermansia* in the intestinal flora to maintain glucose homeostasis. Meanwhile, the hepatic portal vein allows butyrate to get through the epithelial barrier and reach the liver. As an important organ for metabolizing lipids and maintaining cholesterol homeostasis, the liver can regulate lipid metabolism disorders in db/db mice and improve adipose tissue metabolism to regulate lipid levels. The results of LEfse analysis showed that *Proteus*, an opportunistic pathogen, was the dominant flora in the DB group. Additionally, *proteus* was shown to have a correlation with a number of different factors, including body weight, LDL-C, OGTT, IL-1β, IL-6, and TNF-α. Among them, Positive correlations were found with OGTT, IL-1β, and IL-6 (*p* < 0.01), while substantial correlations were seen with TNF-α (*p* < 0.001). *Enterobacteriaceae* was the dominant flora in the IO group of mice, and the evolutionary branching diagram showed that *Enterococcaceae* was similar to DM group. It has been found that *Enterococcaceae* have anti-inflammatory activity, hypocholesterolemic effects, and the capacity to avoid or treat specific illnesses (60). The above study suggested that IO can alleviate inflammation in db/db mice by regulating Proteus and *Enterococcaceae* abundance.

## Conclusions

**Figure 8:**
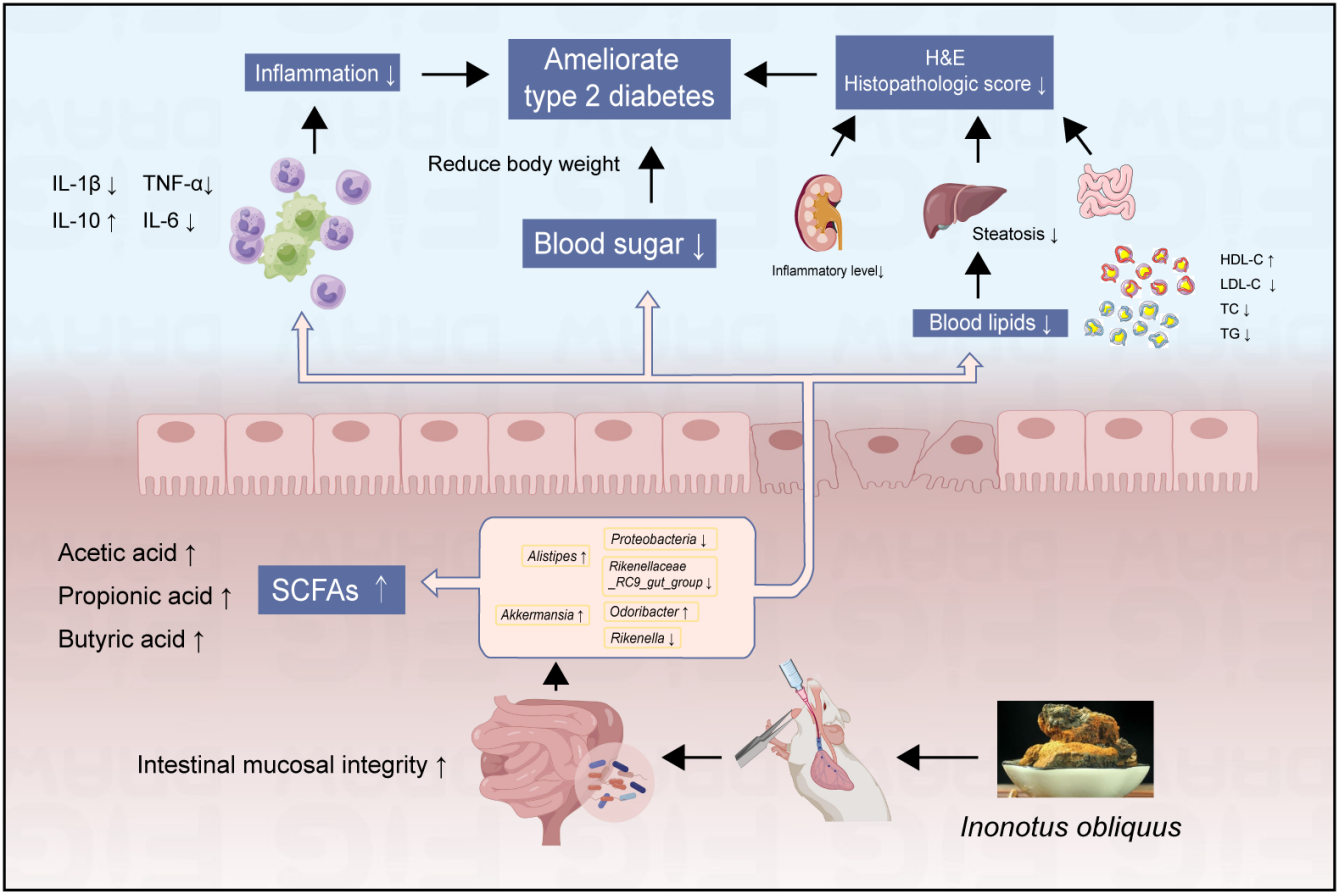
The effects of *Inonotus obliquus* on db/db mice.

These studies showed that *Inonotus obliquus* could effectively reduce body weight and fasting blood glucose and alleviate the extent of lesions in the intestine, liver, renal, and pancreatic organs of diabetic mice (**Figure 8**). Compared with the DB group, treatment with *Inonotus obliquus* effectively changed the intestinal flora composition of db/db mice and improved the intestinal microecological disorders. The mice in the IO group had higher and regulated SCFAs content and alleviated T2DM symptoms by regulating the population density of some bacteria that produce acid such as *Alistipes* and *Akkermansia*.

Meanwhile, *Inonotus obliquus* down-regulated the amount of potentially dangerous bacteria such as *Proteobacteria* and *Rikenellaceae_RC9_gut_group*, and increased the prevalence of good bacteria like *Rikenella* and *Odoribacter*. The glucose homeostasis, blood lipid and inflammation levels, and the degree of organ lesions were further improved in db/db mice (**Figure 8**). Thus, there is a reciprocal relationship of action, *Inonotus obliquus* can keep the intestinal flora in homeostasis by regulating the abundance of intestinal flora, which in turn can control inflammation and alleviate T2DM-related symptoms. In addition, the controlled symptoms of T2DM lead to maintaining the intestinal flora to keep the gut microbes healthy. This offers the possibility to alleviate T2DM symptoms and a series of characteristic complications caused by T2DM by pharmacological regulation of the intestinal flora.

## Data availability statement

The datasets presented in this study can be found in online repositories. The names of the repository/repositories and accession number(s) can be found below: https://www.ncbi.nlm.nih.gov/, No. PRJNA902960.

## Ethics statement

The animal study was reviewed and approved by ethics committee of Zhejiang Academy of Agricultural Sciences (No. 2021ZAASLA83).

## Author contributions

XY and WL contributed to the study design. XY, KW, LX, YC, JN and WZ conducted animal experiments. KW and LX analyzed the samples and data. All authors contributed to the article and approved the submitted version.
