# Supplementary material for: Methanol extract of Inonotus obliquus improves type 2 diabetes mellitus through modifying intestinal flora
Source: Front Endocrinol (Lausanne). 2023 Jan 6;13:1103972. doi: 10.3389/fendo.2022.1103972 (PMC9852891; doi:10.3389/fendo.2022.1103972)
Supplement: Supplementary file 1 [file Table_1.docx]

**Supplementary materials**

Table S1 The intestine pathological criteria

| score | degree of inflammation | inflammatory cell infiltration | degree of damage to the crypts | Crypt abscess | submucosal edema | loss of goblet cells | degree of crypt epithelial hyperplasia |
| --- | --- | --- | --- | --- | --- | --- | --- |
| 0 | Normal | Normal | Normal | Normal | Normal | Normal | Normal |
| 1 | mucosal epithelium + lamina propria | single lesion | base 1/3 of crypt | single lesion | single lesion | single lesion | single lesion |
| 2 | muscularis mucosa | multiple lesions | base 2/3 of crypt | multiple lesions | multiple lesions | multiple lesions | multiple lesions |
| 3 | submucosa | Diffuse | the whole crypt |  | Diffuse | Diffuse | Diffuse |
| 4 | muscular layer + serosa |  | Crypt injury + ulcer |  |  |  |  |

Table S2 The liver pathological criteria

| score | Fibrosis | Ballooning | Lobular Inflammation | Portal Inflammation | Steatosis |
| --- | --- | --- | --- | --- | --- |
| 0 | None | None | No foci | None | <5% |
| 1 | Perisinusoidal or periportal fibrosis | Ballooning | <2 foci per 20× field | Mild | 5% to 33% |
| 2 | Perisinusoidal and periportal fibrosis | Many | 2-4 foci per 20× field | More than mild | 33% to 67% |
| 3 | Bridging fibrosis |  | >4 foci per 20× field |  | >67% |

| score | Mesangial cell proliferation | Matrix broadening | Change of hardening | Crescent bodies or fibers form |
| --- | --- | --- | --- | --- |
| 0 | None | Nomal | Nomal | Nomal |
| 1 | Mild | Segmental widening, no obvious capillary stenosis atresia | Focal segmental distribution,＜30% sclerotic glomeruli | ＜25% segmented focal distribution |
| 2 | Medium | Diffuse widening, ＜50% capillary stenosis atresia | 30%-60% sclerotic glomeruli | 25%-50% diffuse segmental distribution |
| 3 | Severe | Diffuse widening, ＞50% capillary stenosis and atresia | ＞60% sclerotic glomeruli | ＞50% diffuse focal distribution |

Table S3 The glomerular pathological criteria

Table S4 The renal tubular pathological criteria

| Score | Degeneration / Necrosis | Atrophy | Interstitial inflammatory cells infiltrate | Interstitial fibrosis |
| --- | --- | --- | --- | --- |
| 0 | None | None | None | None |
| 1 | Mild | Small focal | Small focal | Small focal |
| 2 | Medium | Slice focus | Conspicuous or focal | Conspicuous or focal |
| 3 | Severe | Diffuse severe atrophy | Diffuse | Diffuse |

Table S5 The abbreviation

| Full name | Abbreviation |
| --- | --- |
| *Inonotus obliquus* methanol extract | IO |
| Type 2 diabetes mellitus | T2DM |
| Ultra-high-performance liquid chromatography-Q exactive mass spectrometry | UHPLC-QE-MS |
| Hematoxylin-Eosin staining | HE |
| Fasting Blood Glucose | FBG |
| Glucose Tolerance Test | OGTT |
| Total Cholesterol | TC |
| Triacylglycerol | TG |
| High-density lipoprotein cholesterol | HDL-C |
| Low-Density Lipoprotein Cholesterol | LDL-C |
| Short-Chain Fatty Acid | SCFA |
| Tumor necrosis factor-α | TNF-α |
| Interleukin-1β | IL-1β |
| Interleukin-6 | IL-6 |
| Interleukin-10 | IL-10 |
| Gas chromatography | GC |
| Oral bioavailability | OB |
| Drug-likeness | DL |
| Traditional Chinese Medicine Systems Pharmacology | TCMSP |
| LDA Effect Size | LEfSe |
| Activating 5'AMP-activated protein kinase | AMPK |
| Nitric oxide synthase | NOS |
| Ursodeoxycholic acid | UDCA |
| Oral glucose insulin sensitivity | OGIS |
